# Patients Seeking Retreatment after Community Paramedic Assessment and Treatment: Piloting a Community Paramedic Unit Program in Southwest Finland

**DOI:** 10.3390/nursrep10020010

**Published:** 2020-11-13

**Authors:** Joonas Hänninen, Anne Kouvonen, Hilla Sumanen

**Affiliations:** 1 RDI Sustainable Wellbeing, South-Eastern Finland University of Applied Sciences, 48220 Kotka, Finland; 2 Faculty of Social Sciences, University of Helsinki, 00014 Helsinki, Finland; anne.kouvonen@helsinki.fi (A.K.); hilla.sumanen@xamk.fi (H.S.); 3 Centre for Public Health, Queen’s University Belfast, Belfast BT7 1NN, UK; 4 Department of Healthcare and Emergency care, South-Eastern Finland University of Applied Sciences, 48220 Kotka, Finland; 5 Faculty of Medicine, University of Helsinki, 00014 Helsinki, Finland

**Keywords:** community paramedic, emergency medical services, prehospital care, ambulance, emergency department

## Abstract

Community paramedic (CP) units are becoming more popular in enhancing a person’s access to the need for care assessment and treatment in acute but non-life-threatening health issues. Simultaneously CP units can reduce the strain on emergency departments (EDs) by treating patients effectively at home. The efficacy of CP units is proven in previous studies, but the details of conditions patients seek retreatment at the ED after a CP unit visit are largely unknown. This study aimed to categorize CP unit patients (*n* = 229) seeking retreatment after a CP unit visit and investigate links between CP unit actions and patients seeking retreatment. The study was based on a data set from a six-month CP unit pilot program in Finland. The main results show that 82% of the patients assessed and treated by the CP unit did not seek retreatment. Low back symptoms and nausea were the main problems patients presented to the ED within 96 h after the CP visit. On-call physician consultation (*p* = 0.335) or CP unit treatment time (*p* = 0.629) were not associated with the frequency of ED presentation. Further studies are needed in order to investigate which types of emergency medical missions are the most suitable for CP units. The findings of this study support the effectiveness of community paramedicine programs.

## 1. Introduction

Community paramedic (CP) units are becoming increasingly popular in the emergency medical services (EMS) setting in Finland. CP units were enabled in Finland by EMS legislation changes in 2018 that allowed a single paramedic nurse to form an EMS unit [1]. Nearly all of Finland’s 21 hospital districts have either operating CP unit(s) or have examined their potential in their operating environment. Due to different operating environments (urban/rural), population density and local health care service level and availability, hospital districts have slight operating variations in community paramedicine programs. The common feature, however, is that the CP units aim to provide the need for care assessment to persons that have difficulties seeking these services otherwise in acute, but not life-threatening, situations and have contacted the emergency response center (ERC) or hospital call center for advice.

Although CP units are new in Finland, community paramedicine programs have been implemented since the 1990s. These early community paramedicine programs addressed the health care needs of underserved rural populations by providing improved access to health care for these communities. Paramedic scope of practice was expanded in these programs to provide chronic disease surveillance, community health education and disease prevention. The paramedics operated with extended medication sets and performed simple procedures such as suturing lacerations [2,3].

From the early rural models, the community paramedicine programs have expanded to urban settings and to cover a broad variety of activities that widen the role of paramedics. These include services associated with primary, social and preventive care, as well as community health interventions. Today the wide scope of community paramedicine is still to increase access to basic health services but also to reduce the need for patient transport and hospital readmissions [2]. The community paramedicine programs are always tailored to address the specific needs of the local community and the local health care system [4]. Local tailoring makes community paramedicine programs agile but hamper the generalization of the methods and results and complicate the research attempts on community paramedicine.

The research published on community paramedicine is largely descriptive in nature and focuses on reporting findings of local community paramedicine programs. In the literature review by Bigham et al. (2013) only one large randomized controlled trial (RCT) was discovered, others being surveys on community paramedicine as a phenomenon or studies of CP units’ patients and their perception of the CP unit model of care [4]. The main results in 10/11 articles are favorable to community paramedicine when compared to the standard EMS, although the evidence was mainly thin. The CP units were able to better provide treatment to patients at home, reducing emergency department (ED) usage [5], and received good customer satisfaction scores [6,7,8]. The safety and effectiveness of CP units were identified as key research points for further research [2,4].

The effectiveness of CP units is often viewed in terms of reduced ED attendances by assessing and treating minor conditions at the patient’s home. In the RCT by Mason et al. (2008) 11% of CP unit patients had an unplanned ED attendance within seven days of the initial episode, and 74% of these patients sought retreatment with a condition related to the initial episode [5]. In the Swedish trial by Magnusson et al. (2015), 38% of the patients stayed at the scene after assessment and treatment by the CP unit. Nineteen percent of these patients presented to the ED within 72 h with residual symptoms [9].

The conditions patients seek retreatment at the ED after a CP unit visit are largely unknown. Vertigo, abdominal pain and unspecified condition/proneness to fall were the main causes of ED retreatment in the Swedish study [9]. In the RCT by Mason et al., the cause of the ED retreatment was not specified [5].

To our best knowledge, there are no studies investigating the associations between the CP unit assignment time and ED attendance or on-call ED physician telephone consultation by the CP and ED attendance. Moreover, there is a paucity of research on the conditions requiring ED attendance after the CP unit visit.

In order to collect experience in a novel community paramedicine pilot program and form a model of operation for future use, the CP unit operated in Turku, Finland, for six months. The aim of this study was to investigate:

(1) How many patients sought ED retreatment for the same symptom/problem after being assessed and treated by the CP;
(2) Which International Classification of Primary Care (ICPC-2)-classified patient groups sought retreatment the most;
(3) If the duration of care provided by the CP unit was longer or shorter in the case of patients who requested retreatment in the ED within the following 96 h; and
(4) If the on-call physician consultation reduced patient ED retreatment within the following 96 h?

## 2. Materials and Methods

### 2.1. The Community Paramedic Pilot Program

The CP pilot program was run by the EMS of the Hospital District of Southwest Finland. The pilot unit operated from May 2018 to October 2018 and took place in the city of Turku and the surrounding urban area (population 290,000). The unit operated in shifts of 12 h a day every day of the week and was based in the Turku University Hospital.

**Figure 1:**
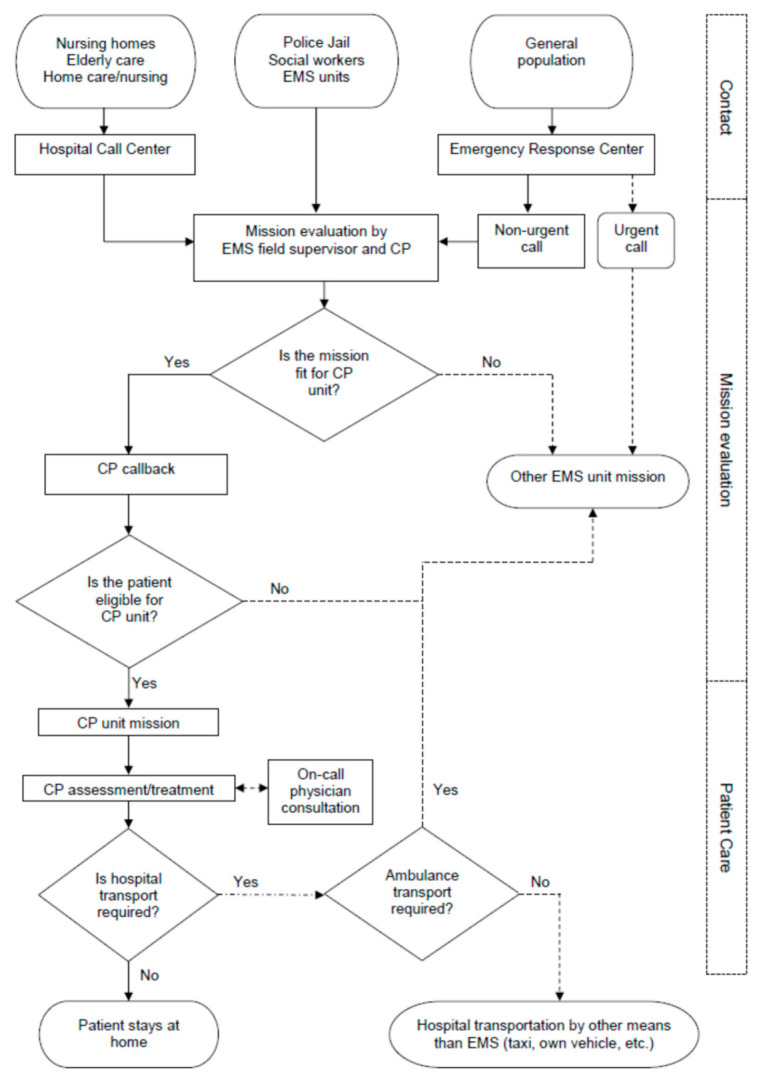
Community paramedic unit mission progression. EMS: emergency medical services; CP: community paramedic.

The main function of the CP unit was to assess the need for treatment, to begin the treatment of the patient in non-urgent calls and to support other EMS units in non-urgent patient encounters that require special examinations, treatments or medication that the CP unit had the capability to deliver. The CP unit was activated by (1) a call to the university hospital call center, (2) by other authorities through the EMS field supervisor or (3) a non-urgent call to the ERC (Figure 1). The CP unit paramedics were experienced advanced-level paramedics (Bachelor’s degree). They received a one-week training course in community paramedicine prior to the pilot, as the CP unit was equipped with a wider medication set and point-of-care diagnostic equipment than a regular ambulance in the area, for example, blood analyzer (i-STAT system), urine test strips, C-reactive protein (CRP) quick analyzer and carboxyhemoglobin (SpCO) monitor. The unit was also equipped accordingly to allow the paramedic to suture or glue small wounds, solve problems with suprapubic or other urine catheters and manage nosebleed (epistaxis) with gelatin foam tamponade. The CP had an option to consult an on-call physician if needed both for decision-making in assessment or to discuss the treatment plan for the patient.

On every mission, the CP had an option to call back to the person in need of help. On missions initiated by the ERC, the CP elaborated the initial assessment by the ERC on the caller’s symptoms. For the missions initiated by the hospital call center or the EMS field supervisor, the CP contacted the original caller for detailed information on the mission and to ensure its suitability for the CP unit.

The CP unit canceled a notable proportion (*n* = 485) of its initiated missions. The main reason for cancellation was that the CP or the EMS field supervisor deemed the mission unfit for the CP unit based on the callback. Reasons for declaring mission unfit for the CP unit were not rigorously documented but included, for example, a clear need for ED treatment defined in the callback, anticipated work safety risk for the CP (intoxication of the patient or others present at the scene, the patient’s known history of violent behavior, etc.) or need for a two-paramedic unit to, e.g., safely provide help for getting up after a fall. In these cases, CP informed the ERC to redirect the mission to a regular EMS unit.

For every accepted CP unit mission, the CP physically assessed the patient at his or her home or other location. Providing assistance solely over the telephone was not considered a thorough enough assessment from the regulatory and legal point of view because CPs are not trained for telephone triage.

All CP unit patient encounters were categorized using the International Classification of Primary Care (ICPC-2) classification system. The ICPC-2 classification is developed for primary care and for use at care pathway starting points such as emergency care. ICPC-2 can be used to classify a patient’s reason for encounter, the problems managed or health care interventions [10].

After the initial assessment and treatment, the CP had the option to give the patient advice for home care or refer the patient to the hospital for further care. The CP model was designed with the intent to treat as many patients at home as it is safe and possible. The model included the possibility for the on-call physician to write an e-prescription for additional medicines to be collected from a pharmacy by the patient. The CP had also the option to refer the patient to the ED for further care. If the transportation did not require the ambulance crew to continue patient monitoring and care on the way, an alternative transport option was arranged with the patient. The CP unit vehicle did not have an option to transport patients.

### 2.2. Data Collection

**Figure 2:**
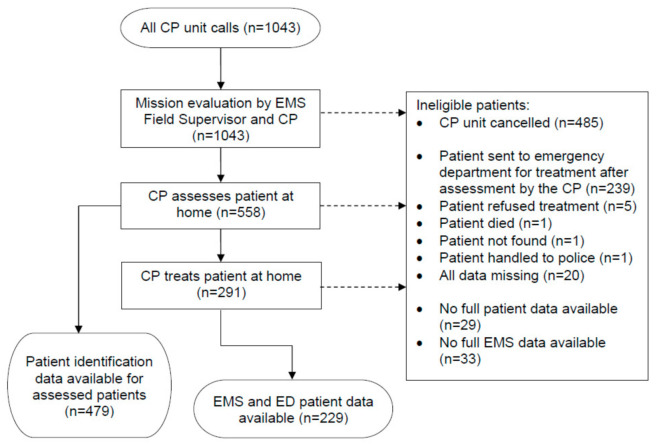
Data collection process. ED: emergency department.

Data collection started with identifying all CP unit calls (*n* = 1043) for missions where the CP was able to assess and treat the patient at home and the patient avoided immediate ED visit. The data included calls from all sources (ERC, hospital call center and EMS field supervisor). These data were merged with the EMS data using the patient’s national ID number (ID) and ICPC-2 classification. The CP unit assessed 558 patients during the pilot program, of which patient identification data were available for 479 patients. The final data set of home-treated patients with ED records available comprised of 229 CP unit missions (Figure 2).

### 2.3. Ethical Considerations

This study used solely secondary data retrieved from administrative registers. Conventions of good scientific practice, data protection and information security were applied. The research permit was granted by the Hospital District of Southwest Finland. The study was based on administrative record data, and ethics approval was thus not required according to the Finnish law [11].

### 2.4. Statistical Methods

For the analysis of patient retreatment rate, patient ID was used for the ED patient record search to identify the patients who had sought retreatment again in 96 h after the CP unit visit for the same reason (classified with same ICPC-2 or same/similar keywords found in the CP unit and ED patient records). A similar patient selection approach was used by Mason et al. (2008) to determine whether the index episode managed by the paramedic was related to the subsequent hospital attendance [5]. The delay for retreatment was calculated using patient record timestamps of the CP unit leaving the patient and the patient admission to the ED. The patients were classified into two groups: “sought retreatment” and “no retreatment”. Some patients sought treatment in 96 h for other reasons than the one they initially presented at the CP unit. This patient group was considered to have received sufficient treatment for their initial condition by the CP and were therefore included in the “no retreatment” group. Some patients were visited by the EMS for the second time at their home because of the same symptom/problem but stayed home after the visit. These patients were also included in the “no retreatment” group, as the study concentrated on ED visits.

Patient ICPC-2 classification was cross-referenced with “sought retreatment” group data using cross-tabulation to discover those ICPC-2 patient groups that sought retreatment the most.

The patient treatment timeframe was extracted from the EMS data using the CP unit timestamps “arriving at the scene” and “leaving the scene” and calculating the patient treatment time from these values. Minimum, maximum, mean and median values were calculated for both “sought retreatment” and “no retreatment” groups for comparison. Statistical relationships between the groups were tested with the Mann–Whitney U-test.

The records of the on-call physician consultation done by the CP were examined using patient records. Patients were dichotomized into two groups: “consultation done” and “no consultation”. The dichotomized physician consultation groups and “sought retreatment” and “no retreatment” groups were cross-tabulated for comparison. A chi-square test was run to evaluate intergroup associations.

SPSS version 26 (IBM Corp., Armonk, NY, USA) was used for the analysis.

## 3. Results

As planned, the CP unit mostly provided assessment and treatment services to elderly patients with minor conditions. The patients assessed by the CP (*n* = 479) were mostly female (60%) and aged between 16 and 100 years (mean age 74.5, median age 80 years and SD 18.1 years). Among these patients, the five most common EMS mission codes for the CP unit were general weakness (23%), pain in spine/torso/limb (19%), vomiting/diarrhea (11%), fall (9%) and arrhythmia/vital function disruption (other) (8%). Ninety-five percent of the CP unit calls were inside Turku city limits, most being inside a 5-km range from the station of the CP unit.

After the CP unit assessment and treatment, 229 of 479 (48%) patients stayed at home. Of these, 187 patients (82%) did not seek retreatment from the ED for the same symptom/problem within 96 h after the CP unit visit. Among these 187 patients, for four patients (2%), the EMS visited the second time at their home within 96 h after the initial CP visit because of the same symptom/problem, but the patient stayed at home. In addition, six patients sought ED treatment within 96 h after the CP unit visit for some other reason than the one they had presented at the CP unit.

In the “sought retreatment” group, 19 patients (8%) sought retreatment from the ED themselves for the same symptom/problem and 19 patients (8%) were transported to the ED by ambulance for the same symptom/problem as the CP unit visit within 96 h.

A quarter of the patients who sought retreatment in the ED had done so in less than six hours after the CP unit had left the scene. The median time for seeking retreatment was 22.5 h, and the mean time was 31 h.

The ICPC-2 class groups including five or more patients and their respective retreatment rates are presented in Table 1. Among these groups, patients with low back symptoms, nausea, nosebleed/epistaxis, general or multiple sites pain, fear of other disease, chest pain or back syndrome with radiating pain sought retreatment from the ED more often than average.

Among the patients who stayed at home after the CP unit assessment and treatment, the mean treatment time period was 1 h 5 min (median 1 h 3 min, range 26 min to 2 h 17 min). Among patients who sought retreatment in 96 h, the mean of the initial treatment time period was 1 h 6 min (median 1 h 4 min, ranging from 26 min to 2 h 12 min). The associations between CP unit treatment time and ED retreatment were not statistically significant (*p* = 0.629).

An on-call physician consultation was done among 193 (84%) of the patients, who stayed at home after the CP unit assessment and treatment. Of those, 34 patients (18%) sought retreatment within 96 h. The associations between on-call physician consultation and ED retreatment were not statistically significant (*p* = 0.335).

## 4. Discussion

In this study, we examined patients who sought retreatment after the CP unit assessment and treatment. The main results show that (1) eighteen percent of the CP unit patients sought retreatment in 96 h, (2) lower back symptoms and nausea were the most common reasons for patients to seek retreatment after the CP unit assessment, (3) CP unit treatment time on-site was not associated with the number of patients seeking retreatment and (4) on-call physician consultation was not associated with the number of patients seeking retreatment.

This study showed that 82% of the patients assessed and treated by the CP unit did not seek retreatment. This finding supports the effectiveness of the community paramedicine program. The patient retreatment rate (18%) can be considered low and is similar to Mason’s (11%) [5] and Magnusson’s (19%) [9] results. Due to differences in the characteristics of patient groups, the results might not be directly comparable. In the study by Mason et al. [5], 88% of the patients presented after a fall, whereas in our study general weakness was the main complaint.

The most common reasons for retreatment differed from those identified in a previous study. In the study by Magnusson et al. (2015), the most common reasons to seek ED care within 72 h of the CP visit were vertigo/headache, abdominal pain and unspecified condition/proneness to fall (8/7/6 patients/group, respectively) [9]. The limitations in that study and in our study are very small retreatment patient groups, <10 patients, which makes comparison among them pointless and warrants further studies with larger data sets.

The results show that three-quarters of the patients the CP unit visited were over the age of 65. This was anticipated in the planning of the unit, which was done in close collaboration with the geriatric services of the city of Turku. This is also in line with the study by Mason et al. (2007), where the mean age of patients was 85.5 years. However, the inclusion criterion for that study was age >65 years [12].

In Magnusson’s (2015) study [9], the mean age of patients was 62.2 and the median age 68 years, which also indicates that CP programs target mainly older people. These findings should be taken into consideration when planning future community paramedicine programs.

As far as we are aware, the timeframe in which patients sought retreatment either from a hospital ED or by recalling an ambulance has not been investigated before in the CP unit context. Our study showed that a quarter of the patients in the Turku CP pilot program who sought retreatment after being assessed and treated by the CP unit did so in less than six hours. This could indicate suboptimal care, but it could also indicate that a treatment plan was made for the patient to deliberately continue care later in the ED after initial assessment and treatment done by the CP.

The strength of this study is that the data set used comprised all CP unit missions during the six-month pilot program. The data were collected from patient and EMS records that are considered reliable sources. However, 14% of the EMS data and 6% of the total data was incomplete and had to be discarded during the data collection process.

The weakness of this study is the small number of patients who sought retreatment after the CP unit visit. Thus, the final data set of the patients assessed and treated at home by the CP was not large enough for making further conclusions.

In addition, the data collection process of this study showed that the directing of EMS missions to CP units could be further developed. The CP unit was canceled on 47% of the missions either by the CP calling back to the patient for a detailed patient condition interview or due to a presumed safety risk for the single working CP. This suggests that selecting the missions suitable for CP units could be planned more efficiently.

The study design may cause some patients to seek retreatment outside the reach of this study. Apart from the ED, patients may also seek retreatment for minor conditions from local public health centers or private clinics. This, however, is outside the scope of this study as the decision was made to focus solely on ED retreatment rates. The decision of placing the cutoff point to 96 h for the tracking of CP patients seeking ED retreatment may have caused data loss if patients chose to seek ED retreatment for the same reason after 96 h. However, the results show that patients seek retreatment commonly on the same or the next day after the CP visit. This mitigates the possibility of lost patients due to too short tracking times.

Due to differences between EMS systems, the results of this study may not be generalizable to other countries. Nevertheless, globally EMS and EDs are burdened by the increase of patients, and CP units may provide a partial solution when adapted to the local healthcare system.

## 5. Conclusions

The vast majority of the patients did not seek retreatment within 96 h after the CP unit visit. Overall, the current evidence supports community paramedicine programs, but further studies are needed in order to investigate which types of EMS missions are the most suitable for CP units. Increasing evidence on this would support further development of CP programs in a cost-effective way.

## Figures and Tables

**Table 1:** The CP unit visits by the ICPC-2 class and patient group retreatment rates in the ED within 96 h.

ICPC-2 Class	Missions, *n* (%)	Retreatment in 96 h, *n* (%)
A04 Weakness/tiredness general	30 (13)	3 (10)
L03 Low back symptom/complaint	26 (11)	7 (27) ^1^
D09 Nausea	12 (5)	5 (42) ^1^
A29 General symptom/complaint other	11 (5)	1 (9)
N17 Vertigo/dizziness	10 (4)	1 (10)
R06 Nosebleed/epistaxis	9 (4)	3 (33) ^1^
K80 Cardiac arrhythmia NOS ^2^	9 (4)	1 (11)
A01 Pain general/multiple sites	8 (3)	3 (38) ^1^
A27 Fear of other disease NOS ^2^	7 (3)	2 (29) ^1^
D01 Abdominal pain/cramps general	6 (3)	2 (33) ^1^
A11 Chest pain NOS ^2^	5 (2)	1 (20) ^1^
L86 Back syndrome with radiating pain	5 (2)	1 (20) ^1^
Other symptoms/problems	91 (40)	8 (9)
Total	229	38 (17)

^1^ Higher than average (>17%); ^2^ NOS: not otherwise specified.
